# Predictors of neovascular activity during neovascular age-related macular degeneration treatment based on optical coherence tomography angiography

**DOI:** 10.1038/s41598-019-55871-8

**Published:** 2019-12-17

**Authors:** Kunho Bae, Hyo Jung Kim, Yong Kyun Shin, Se Woong Kang

**Affiliations:** 1 Department of Ophthalmology, Dongguk University, Ilsan Hospital, Goyang, South Korea; 2 Department of Ophthalmology, Samsung Medical Center, Sungkyunkwan University School of Medicine, Seoul, Korea

**Keywords:** Macular degeneration, Prognostic markers, Macular degeneration

## Abstract

The advent of anti-vascular endothelial growth factor (VEGF) therapies has remarkably improved the functional outcomes of neovascular age-related macular degeneration (nAMD) patients. However, there are guidelines on how to start treatment, the guidelines for discontinuing treatment are not yet clear. In this respect, the treat-extend-stop (TES) protocol have showed us the possibility of discontinuing treatment. In this study, we tried to investigate optical coherence tomography angiography (OCTA) biomarkers related to recurrence of neovascular activity in eyes with nAMD undergoing treatment using TES protocol. A total of 134 eyes with nAMD were divided into two groups (stop, non-stop) depending on whether they met criteria for stopping anti-VEGF treatment. Quantitative and qualitative OCTA parameters including the morphologic pattern of choroidal neovascularization (CNV) were compared between groups. Of these, 44 eyes (32.8%) were in the stop group and 90 eyes (67.2%) were in the non-stop group. In multivariate regression analysis, closed-circuit pattern of CNV and the presence of peripheral loop were associated with the non-stop group (all p* < *0.001). Our results imply that the morphologic appearance of CNV on OCTA after anti-VEGF treatment may be a useful biomarker to predict weaning from treatment.

## Introduction

Age-related macular degeneration (AMD) is the leading cause of irreversible blindness in people 50 years of age or older in industrialized countries and affects a large proportion of the elderly^1–3^. Although neovascular AMD accounts for only two-thirds of prevalent late-stage cases, it is responsible for over 80% of severe vision loss and blindness attributable to AMD^2,4^. Angiogenesis is the major mechanism promoting the progression of neovascular AMD^5,6^. and the advent of anti-vascular endothelial growth factor (VEGF) therapies has remarkably improved the functional outcomes of patients^7^. However, treatment effectiveness depends on the dose regimen and is variable^8^. Although some clinical trials have shown improvements in visual outcomes with both fixed dosing and pro re nata (PRN) dosing regimens^4,7,9^, the long-term outcomes of PRN are reported to be significantly worse than those of fixed dosing^10^. Recently, treat-and-extend regimens, along with their variant, the treat-extend-stop (TES) protocol, have been reported to achieve comparable efficacy to fixed dosing^11,12^. Because extended suppression of VEGF may induce atrophic degeneration^13^. the TES regimen exhibits potential for establishing effective treatment strategies to avoid anti-VEGF overtreatment and reduce treatment burden as well.

The spectrum of disease associated with AMD has expanded with the advances in diagnostic technologies, enabling clinicians to better differentiate unique CNV subtypes, such as polypoidal choroidal vasculopathy (PCV) which is now recognized as being more common among Asian patients^14–17^. Polypoidal choroidal vasculopathy has been shown to have similarities and differences compared with typical neovascular AMD in genetic predisposition, clinical and pathologic characteristics, and response to photodynamic therapy (PDT) or anti–VEGF agents; whether PCV is a subtype of AMD or a different clinical entity remains controversial^18^. Choroidal neovascularization (CNV) plays a key role in the pathogenesis of typical AMD and PCV, which involves the growth of new blood vessels that originate from the choroid through a break in the Bruch’s membrane into the sub–retinal pigment epithelium or subretinal space. Fluorescein angiography (FA), indocyanine green angiography (ICGA), and optical coherence tomography (OCT) are the gold standard diagnostic tools used to detect and evaluate CNV^19^. More recently, the introduction of OCT angiography (OCTA) has provided a noninvasive system to better assess the microvascular morphology of these lesions^20,21^. The morphologies of CNV associated with neovascular AMD have been extensively described using OCTA, including “sea fan,” “medusa,” “glomerulus-shaped,” “pruned tree,” “vascular loop” and long-term growth patterns^22–24^. In addition, qualitative and quantitative OCTA biomarkers of CNV have been investigated^21,25^. However, morphological patterns vary between the reports. Furthermore, no previous report has sufficiently addressed the relationship between CNV morphology on OCTA and neovascular activity.

In this study, we aimed to investigate the relationships between en face OCTA morphology and neovascular activity of CNV after anti-VEGF treatment, with the goal of predicting the feasibility of discontinuing treatments.

## Results

A total 134 eyes of 128 patients with neovascular AMD were enrolled in this study. The ages of the patients at presentation ranged from 54 to 92 years (mean 73.8 years) and there were 83 (61.9%) male patients. Of these, 44 eyes (32.8%) met the criteria for treatment cessation (stop group), with a mean overall follow-up of 62.8 months.

Table 1 outlines the baseline characteristics of both groups. The mean age in the stop group was 73.0 years (range: 56–90 years). The multimodal imaging approach diagnosed polypoidal choroidal vasculopathy in 27 eyes (61.4%) and typical exudative AMD in 17 eyes (38.6%). Presence of subretinal fluid (SRF) was detected in 28 eyes (64.6%), and inner retinal cyst (IRC) in 13 eyes (29.6%). The mean visual acuity at baseline was 20/63 (0.50 in logMAR). In the non-stop group, the mean age was 74.1 years (range: 54–92). The polypoidal choroidal vasculopathy was present in 60 eyes (66.7%), while typical exudative AMD was present in 30 eyes (33.3%). Presence of SRF was noted in 75 eyes (85.2%), and IRC in 18 eyes (20.5%). The mean visual acuity at baseline was 20/56 (0.45 in logMAR). Males accounted for 46.5% of patients in the stop group and 70.6% in the non-stop group (p = 0.006). There were no significant differences between the two groups regarding age, duration of follow-up, lens status, type of CNV, or OCT characteristics other than presence of SRF (p* = *0.005). The mean number of treatments, including intravitreal anti-VEGF injections, switching of anti-VEGF agents, and number of PDT was significantly higher in the non-stop group (all p < 0.05).

**Table 1:** Clinical characteristics of patients with age-related macular degeneration managed with a Treat-Extend-Stop protocol.

Parameters	Stop group	Non-stop group	p-value
	n = 44	n = 90	
**Baseline Characteristics**			
Age, yrs	73.0 (56–90)	74.1 (54–92)	0.452
Sex (Male/Female)	20/24	63/27	0.006
Lens (Phakia/Pseudophakia)	39/5	70/20	0.130
Type of CNV (typical AMD/PCV)	17/27	30/60	0.546
BCVA, Snellen (logMAR)	20/63 (0.5)	20/56 (0.45)	0.945
Central subfoveal thickness, μm	367.1 (195–795)	373.3 (213–792)	0.584
Choroidal thickness, μm	217.8 (47–412)	219.2 (56–517)	0.937
Subretinal fluid	28 (64.6%)	75 (85.2%)	0.005
Inner retinal cyst	13 (29.6%)	18 (20.5%)	0.245
**Treatment**			
Anti-VEGF injection, number	11.1 (0–32)	24.1 (16–60)	<0.001
Switching of anti-VEGF agents	6 (13.6%)	32 (35.6%)	0.008
Photodynamic therapy	0.64 (0–4)	1.36 (0–8)	0.036
**Follow-up data**			
Follow-up time, mo	61.1 (24–141)	63.6 (24–141)	0.470
BCVA at last visit, Snellen (logMAR)	20/47 (0.38)	20/54 (0.43)	0.589

BCVA: best-corrected visual acuity; CNV: choroidal neovascularization; PCV: polypoidal choroidal vasculopathy; BCVA: best-corrected visual acuity; VEGF: vascular endothelial growth factor.

Continuous variables are reported as mean (range) values. All other data are n (%).

**Figure 1:**
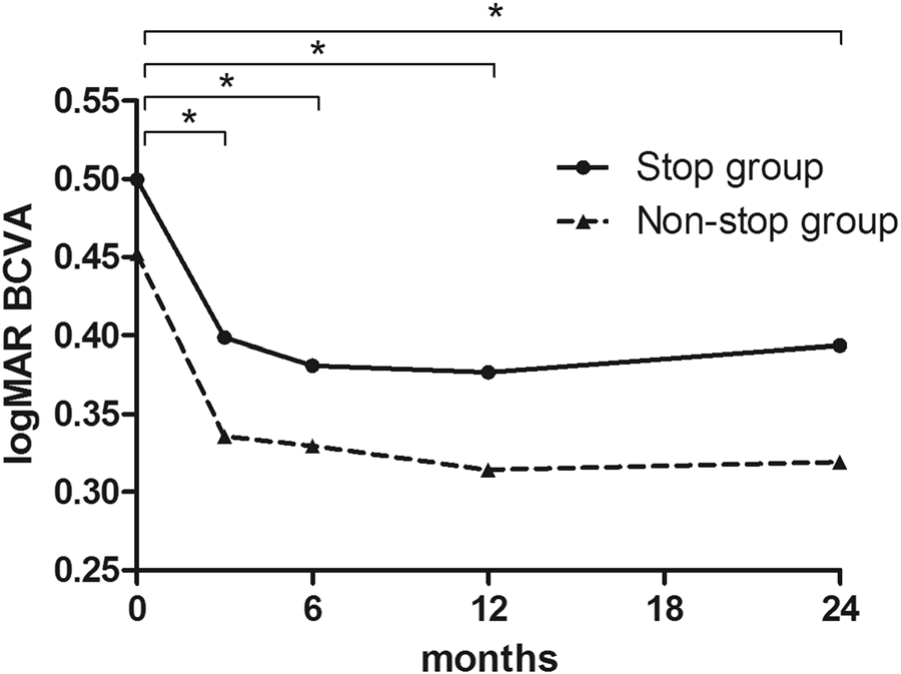
Changes in best-corrected visual acuity (BCVA) of eyes with an age-related macular degeneration managed with a Treat-Extend-Stop protocol at 3, 6, 12, and 24 months after initiating anti-vascular endothelial growth factor treatment. BCVA improved in both groups and showed no significant differences between the two groups according to follow-up period (all p < 0.05, asterisk).

Mean central subfield thickness (CST) values measured at baseline and 3, 6, 12, and 24 months after initiating treatment were 367.1 ± 127.9, 251.0 ± 52.8, 259.2 ± 56.3, 271.4 ± 87.0, and 247.0 ± 41.0 μm, respectively, in the stop group and 373.3 ± 105.6, 268.7 ± 64.9, 281.2 ± 70.3, 273.7 ± 60.0, and 277.0 ± 71.4 μm, respectively, in the non-stop group. There was no significant difference in CST between the two groups up to 12 months. However, at 24 months after treatment, CST was lower in the stop group than the non-stop group (p = 0.037). Mean logMAR BCVA values measured at baseline, and 3, 6, 12, and 24 months after initiating treatment were 0.50 ± 0.44, 0.40 ± 0.45, 0.38 ± 0.46, 0.38 ± 0.45, and 0.39 ± 0.51, respectively, in the stop group and 0.45 ± 0.33, 0.33 ± 0.27, 0.33 ± 0.27, 0.31 ± 0.25, and 0.32 ± 0.27, respectively, in the non-stop group (Fig. 1). The patients in both groups had relatively good baseline initial BCVA and there was a significant improvement during the entire follow-up period (all p < 0.05). However, there was no significant difference in BCVA between the two groups.

### Optical coherence tomography angiography analysis

En face OCTA demonstrated that, of the 90 eyes in the non-stop group, 33 eyes (36.7%) were identified as having open-circuit patterns and 57 eyes (63.3%) were identified as having closed-circuit patterns. In the non-stop group, there were no eyes for which CNV was not detected on OCTA. Of 44 eyes in the stop group, 21 eyes (77.8%) were identified as having open-circuit patterns, 6 eyes (22.2%) were identified as having closed-circuit patterns, and CNV was not observed in 17 eyes (38.6%). There was a significant difference in CNV patterns between the two groups (p* < *0.001). The non-stop group demonstrated capillary fringe and peripheral loop more frequently than the stop group (73.3% vs. 45.4%, p* = *0.002, and 88.7% vs. 11.3%, p < 0.001, respectively). The inter-observer reproducibility showed good agreement with the level of 0.642 (95% CI: 0.529–0.755, p < 0.001), 0.794 (95% CI: 0.740–0.848, p < 0.001), and 0.820 (95% CI: 0.770–0.870, p < 0.001) in the analysis of circuit pattern, capillary fringe, and peripheral loop, respectively.

The mean area of the NV complex was 0.53 mm^2^ in the stop group and 0.76 mm^2^ in the non-stop group (p = 0.001). The total length of the NV was 14.2 mm in the stop group and 20.2 mm in the non-stop group (p = 0.002). The mean number of vessel junctions was 72.9 in the stop group and 103.8 in the non-stop group (p = 0.004). The mean number of NV end points was 54.0 in the stop group and 75.7 in the non-stop group (p = 0.004) (Table 2).

**Table 2:** Optical coherence tomography angiography characteristics of patients with age-related macular degeneration managed with a Treat-Extend-Stop protocol.

Parameters	Stop group	Non-stop group	p*-*value
	n = 44	n = 90	
Area of CNV, mm^2^	0.53 (0–2.0)	0.76 (0.1–1.9)	0.001*
Total length of CNV, mm	14.2 (0–48.8)	20.2 (3.1–51.9)	0.002*
Number of CNV junctions	72.9 (0–232)	103.8 (12–324)	0.004*
Number of CNV end points	54.0 (0–169)	75.7 (10–205)	<0.001^†^
Undetectable CNV	17 (38.6%)	0	<0.001^‡^
Pattern of CNV			<0.001^‡^
Open-circuit	21 (77.8%)	33 (36.7%)	
Closed-circuit	6 (22.2%)	57 (63.3%)	
Peripheral loop	8 (18.8%)	63 (70.0%)	<0.001^§^
Capillary fringe	20 (45.5%)	66 (73.3%)	0.002^§^

CNV, choroidal neovascularization.

Continuous variables are reported as mean (range) values. All other data are n (%).

*Calculated by Wilcoxon rank sum test.

^†^Calculated by two sample t test.

^‡^Calculated by Fisher’s exact test.

^§^Calculated by chi-square test.

In multivariate logistic regression analysis, presence of closed-circuit pattern, presence of peripheral loop, and large number of anti-VEGF injections were independent factors associated with the non-stop group (all p < 0.001) (Table 3).

**Table 3:** Stepwise multivariate logistic regression analysis of factors correlated with continuation of treatment in patients with age-related macular degeneration managed with a Treat-Extend-Stop protocol.

Parameters	Univariate	Multivariate
	p-value	p-value
Pattern of CNV	<0.001	<0.001
Peripheral loop	<0.001	<0.001
Capillary fringe	0.003	0.993
Area of CNV, mm^2^	0.033	0.646
Total length of CNV, mm	0.024	0.955
Anti-VEGF injection	<0.001	<0.001
Switching of anti-VEGF agents	0.011	0.090

CNV: choroidal neovascularization; VEGF: vascular endothelial growth factor.

## Discussion

In our study, we detected differences in qualitative and quantitative features on OCTA in treatment-naïve neovascular AMD patients managed with a TES protocol who were (stop group) or were not (non-stop group) able to stop treatment. While there were no significant differences between the two groups regarding subtypes of CNV and OCT characteristics, we noted a higher prevalence of features (the presence of closed-circuit pattern, peripheral loop, and capillary fringe) associated with sustained neovascular activity in the non-stop group when compared to the stop group. Conversely, in the stop group, CNV was not identified in 38.6% of the eyes, and the presence of an open-circuit pattern, suggesting maturation of neovascular complex, was more frequent. In terms of quantitative measures, the area, total length, and number of junctions of CNV as well as the mean number of intravitreal anti-VEGF injections was higher in the non-stop group. These data suggest that the morphologic appearance of NV may be used to determine the prognosis of anti-VEGF therapy response in patients with neovascular AMD.

In contrast to other OCTA studies, we compared OCTA features according to response to a long-term therapy using a TES protocol. TES, which is a modified treat-and-extend protocol^12^, individualizes treatment intervals based on ongoing assessments of treatment response^26^. The protocol is widely used because of its efficacy for improving vision and reducing the treatment burden. In this study, a total of 32.8% of eyes met the criteria for treatment cessation, with an average follow-up of 63 months. Our findings are consistent with previously-published observations, in which 37.3% of eyes met the criteria for treatment cessation using a TES protocol^27^. In that study, patients managed with a TES protocol were able to successfully stop treatment, but reported that their visual acuity temporarily decreased when the treatment was stopped and the condition recurred^27^. As with the results of PRN regimens, recurrence after discontinuation may worsen long-term visual prognosis^28^. However, relatively few studies examining prognostic biomarkers for suspending anti-VEGF treatment exist^29^.

**Figure 2:**
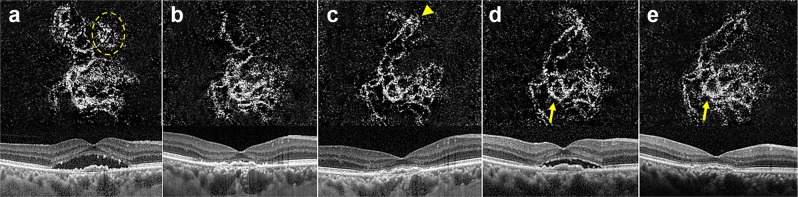
Optical coherence tomography angiography (OCTA) images (3 × 3 mm) with corresponding OCT B-scans of choroidal neovascular lesions in neovascular age-related macular degeneration at baseline (**a**), 4 weeks (**b**), 8 weeks (**c**), 14 weeks (**d**), and 26 weeks (**e**) after undergoing intravitreal aflibercept treatment using a treat-extend-stop protocol. Note the disappearance of immature capillary fringes (dotted circle) after initial anti-vascular endothelial growth factor (VEGF) therapy and formation of peripheral loop (arrow head) in the same location. There were no significant changes in overall morphology after the initial loading phase of anti-VEGF (**d**,**e**), however, dilation of central vessels and formation of anastomosing connections (arrow) intensified during periodic anti-VEGF treatment.

OCTA is a novel, non-invasive imaging technology that provides detailed microvascular morphologic information and information about quantitative features of neovascular lesions in AMD^30^. Previous reports identified several OCTA biomarkers in neovascular AMD^21,24,31,32^, and Coscas *et al*. estimated that different CNV patterns detected on OCTA corresponded with treatment decisions made after multimodal imaging^31^. However, morphologic classifications used in previous studies were complicated or varied among reports, and thus the clinical applications of this information have been limited. In our study, we characterized and analyzed patterns of CNV by dividing our cohort into two categories, which may reflect the periodic process of neovascular complex maturation. Spaide previously proposed that the remodeling process underlying neovascular complex enlargement involves repeated cycles of vascular pruning and reproliferation during the periodic inhibition of VEGF^33^. In that study, after a mean number of 47 injections of anti-VEGF, branching points and many vascular anastomotic connections were observed together with a lack of surrounding choriocapillaris. Anti-VEGF treatment is considered to prune vascular sprouts of CNV, resulting in increased flow through the remaining vessels leading to arteriogenesis, with an additional effect of dilation and ingrowth of vessels (Fig. 2). Regrowth of vascular sprouts may form connections between afferent and efferent vessels, and after a number of cycles, these anastomotic connections enlarge to shunt vessels around the border of the vascular circuit. In this regard, observing CNV with closed-circuit pattern implies that the neovascular complex had undergone remodeling (arteriogenesis and angiogenesis) over a prolonged period. In other words, previous frequent recurrences necessitate repeated treatments and there is a high likelihood that continuous treatments will be needed in the future in such cases.

**Figure 3:**
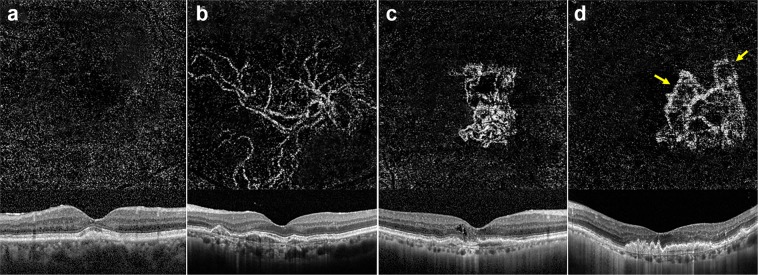
Optical coherence tomography angiography (OCTA) images (3 × 3 mm) with corresponding OCT B-scans of choroidal neovascular (CNV) lesions in age-related macular degeneration. (**a**) En face OCTA image without detectable CNV at 47 months after last treatment. This case was classified in the stop group and received a total of 8 injections of ranibizumab. (**b**) En face OCTA image of an open-circuit pattern patient at 7 months after the last treatment. This case was classified in the stop group and a total of 21 injections of aflibercept were performed. (**c**) En face OCTA image of a closed-circuit pattern patient at 2 months after the last treatment. This case was classified in the non-stop group and a total of 17 injections of ranibizumab were performed, after which the patient was switched to bevacizumab. Treatment was gradually discontinued according to the Treat-Extend-Stop protocol, but the inner retinal cyst recurred every time the treatment interval was extended over 3 months. Note the relatively large portion of anastomotic margin of CNV compared to patients with open-circuit pattern. Inner retinal cyst was observed on OCT B-scans, despite the fact that the patient was currently undergoing maintenance therapy every 2 months. (**d**) Another case with a closed-circuit pattern in the non-stop group. This is an en face OCTA image taken 2 months after the last treatment, after a total of 26 injections of bevacizumab after being switched from ranibizumab. Note the peripheral loop (arrows) as well as the anastomotic margin of CNV. Since the inner retinal cyst recurred when the treatment interval was prolonged for more than 3 months, treatment was administered every 2 months.

**Figure 4:**
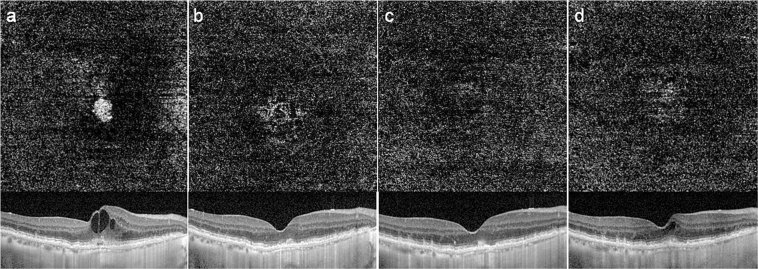
Optical coherence tomography angiography (OCTA) images (3 × 3 mm) with corresponding OCT B-scans of choroidal neovascular lesions in neovascular age-related macular degeneration at baseline (**a**), 4 weeks (**b**), 8 weeks (**c**), and 14 weeks (**d**) undergoing intravitreal aflibercept treatment using a Treat-Extend-Stop protocol. Note the size of the choroidal neovascularization (CNV) decreased during the treatment every 4 weeks and is not detected eventually (**c**). When the treatment interval is extended to 6 weeks, the flow of CNV was detected in OCTA with inner retinal cyst in structural OCT.

In this study, several OCTA biomarkers were identified and correlated with recurrent or sustained exudation of CNV while using a TES protocol, in addition to the closed-circuit pattern of CNV and the peripheral loop showing strong associations with the non-stop group (Fig. 3). Presence of a capillary fringe indicates indistinct tiny capillaries around the border of the vascular circuit, which may be referred to as active neovascularization. Therefore, the coexistence of closed-circuit pattern and capillary fringe signifies sustained VEGF activity over a long period of time that is still ongoing. A significant proportion (38.6%) of eyes among the stop group had no detectable CNV on OCTA, while we observed no cases without detectable CNV on OCTA in the non-stop group. This suggests that if CNV is not observed in OCTA after anti-VEGF treatment, then the treatment may be discontinued. The disappearance of the lesion on OCTA images might be related to both a diminished flow within the CNV after anti-VEGF treatment and to choriocapillaris atrophy (Fig. 4)^34^. Lack of detectable CNV on OCTA after treatment constitutes a specific biomarker indicating no recurrence of exudation after discontinuation of anti-VEGF treatment. We observed a significant difference in the proportion of open-circuit pattern between the two groups (36.7% in non-stop group vs. 77.8% in stop group, except for eyes without detectable CNV) as well as area and total length of CNV. It remains unclear why the morphologic patterns are associated with the discontinuation of treatments. In an open-circuit pattern, anatomical changes after the remodeling process, such as anastomosis with choroidal vessels away from the central fovea, may serve to stop the exudation. Otherwise, morphological patterns may be the result reflecting neovascular activity rather than cause, although intraocular VEGF levels were not assessed in this analysis.

We also verified previously reported principal patterns of CNV: “pruned tree,” “sea fan,” “glomerulus-shaped,” and “medusa”^22,35^. These morphologies provide pathologic correlates of neovascular AMD with distinct pattern characteristics and information about structural response to treatment. However, the substantive morphologic features of neovascularization do not always conform to these categories, and discrepancies in the diverse morphologic classifications used by different examiners make it difficult to determine its clinical significance. In this regard, we devised a more intuitive and simplified classification to reduce discrepancies and facilitate clinical application. Miere and associates indicated that initial CNV patterns frequently switch toward mature patterns after anti-VEGF therapy^35^. In our study, OCTA images were analyzed after long-term treatment and observation, demonstrating relatively stable CNV patterns. It remains unclear how patterns observed at early stages changed over time in our cohort. We suggest further longitudinal studies to verify the phenomenon of switching patterns.

Limitations of our analysis include its retrospective nature and the inconsistent follow-up periods and OCTA assessment among patients. Eyes were analyzed regardless of subtype of exudative AMD, which could reflect real-world outcomes. However, since the Supplemental Table 1 shows that no significant differences exist between the PCV and typical nAMD population in regards to the various OCTA parameters, the influence of AMD subtype is believed to be minimal. In addition, there was no significant difference between the two groups (stop vs. non-stop) regarding the type of CNV. Another limitation of our study is the use of two-dimensional assessments of CNV lesions, although the measurements were calculated using semi-automated software. Only en face area calculations could be performed, as automated volumetric analysis of CNV is not available in current OCTA systems, a limitation shared with other OCTA studies. Additionally, the standardization of treatment regimens may be complicated by variation such as employing different anti-VEGF agents, even while following the same TES protocol. However, the large number of patients included in our sample and long follow-up period make our dataset a valuable addition to the literature. Despite limitations, we believe our study provides useful information for screening patients who may discontinue treatment using the TES regimen.

In conclusion, our results suggest that two types of treatment response to TES may be expected, depending upon morphologic patterns on OCTA. Our findings have two main consequences: first, confirming the validity of TES regimens and promoting the possibility of discontinuing treatment, and second, verifying the morphologic pattern of CNV on OCTA after anti-VEGF treatment as a useful biomarker determining whether to stop anti-VEGF injection or to continue disease suppression by maintenance therapy. All patients without detectable CNV on OCTA were able to cease treatment, while the presence of a closed-circuit pattern along with the peripheral loop, which may signify sustained VEGF activity, tended to indicate the necessity of maintaining treatment. Analyzing patterns of neovascularization and other morphologic features may reduce the incidence of macular atrophy after continuous anti-VEGF injections and the burden of treatment, and provide better individualized follow-up and treatment of neovascular AMD.

## Methods

This retrospective cohort study was approved by the Institutional Review Board of the Samsung Medical Center (IRB no. 2018–11–046) and conducted in accordance with the ethical standards stated in the Declaration of Helsinki. All patients provided written informed consent before the treatment.

### Study population

We reviewed the electronic medical records of patients older than 50 years who were diagnosed with neovascular AMD including PCV at the Department of Ophthalmology, Samsung Medical Center, from January 2007 through September 2018. Patients were identified and included if they had been followed-up for more than 24 months after receiving intravitreal anti-VEGF treatment and undergoing OCTA in at least one eye. All patients were followed and treated by a single retina specialist (S.W.K.). Clinical charts and multimodal imaging including fluorescein and indocyanine green angiography, spectral-domain OCT, and swept-source OCTA were reviewed for each patient and only the eyes with a definite CNV on angiography at baseline were included. Eyes with refractive errors larger than 6 diopters of spherical equivalent or axial length greater than 26 mm, existence of significant media opacity or massive subfoveal hemorrhage, history of ocular inflammation, history of intraocular surgery (other than cataract surgery), history of ocular trauma, or glaucoma were excluded. Patients with loss of vision not related to neovascular AMD were also excluded.

### Treatment protocol

Anti-VEGF agents were administered intravitreally during this study, including ranibizumab (Lucentis; Genentech Pharmaceuticals; San Francisco, CA), aflibercept (Eylea; Regeneron Pharmaceuticals, Tarrytown, NY), and bevacizumab (Avastin; Genentech Pharmaceuticals, San Francisco, CA). The patients received treatment in accordance with a TES protocol, such that after 3 injections at 4-week intervals, the treatment period was extended if the macula was determined to be without fluid on OCT and clinical examination^26^. As soon as the macula was free of fluid, the treatment intervals were increased successively by 2 weeks until 12 weeks had passed. Patients then received 2 or more injections at 12-week intervals, and if the macula remained dry, treatments were suspended and the patients were monitored carefully. If at any point a new CNV developed or recurrence of the previous CNV was found, treatment was reinitiated immediately. Clinical activity of CNV was defined similarly to the retreatment criteria in the pro re nata arm of the HARBOR studies, and included any evidence of disease activity on spectral domain OCT (Spectralis HRA + OCT; Heidelberg Engineering, Heidelberg, Germany) such as presence of intraretinal fluid, subretinal fluid, or sub–retinal pigment epithelium fluid^36^. Active polyps were treated using PDT with verteporfin (Visudyne, Novartis International AG, Basel, Switzerland) guided by ICGA. Full-dose, full-duration PDT was performed.

### OCTA image acquisition and analysis

OCTA images were obtained by a swept-source OCT device (DRI OCT Triton; Topcon Corporation, Tokyo, Japan). The device operated with a central wavelength of 1050 nm, an acquisition speed of 100,000 A-scans per second, and axial and transversal resolutions of 7 and 20 μm in tissue, respectively. The 3 mm × 3 mm scans were obtained with each cube consisting of 320 clusters of four repeated B-scans centered on the fovea. Automatic segmentation was performed by the viewing software to generate en face images of the NV lesion after adjusting the level of the segmented layer on the B-scans to best visualize the NV complex. Two horizontal segmentation lines contoured to the ‘outer retina’ profile were used to ensure clear visualization of the NV complex, extending from the inner plexiform layer/inner nuclear layer to the outer border of the Bruch’s membrane. In cases of segmentation errors, the thickness between the two segmentation lines was manually adjusted to include the whole NV complex. After each acquisition, the scans were reviewed to ensure adequate image quality (Topcon image quality index ≥50) and detect motion artifacts.

**Figure 5:**
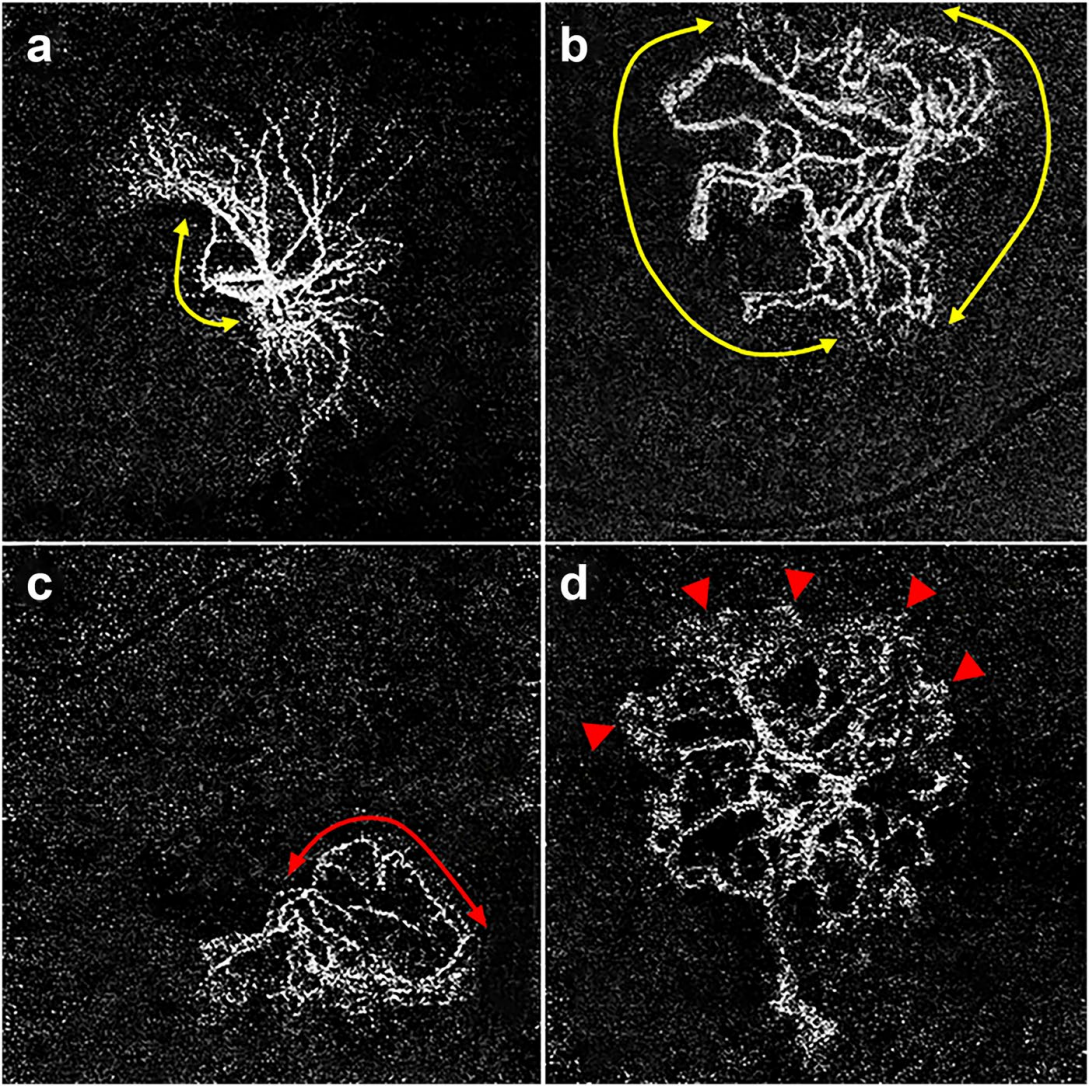
Morphologic patterns of choroidal neovascularization (CNV) on optical coherence tomography angiography were classified into two categories: CNV with an open circuit, and closed circuit. Closed circuit was defined as anastomotic vessels bounding the outer border of the vascular lesion for more than 50% of the entire CNV margin. Note the relatively small portion (less than 50%) of the anastomotic margin (yellow line with arrow heads) included in the open-circuit pattern (**a**) compared to the closed-circuit pattern (**b**). Images were additionally analyzed for presence of peripheral loop (**c**, red line with arrow heads) and capillary fringe (**d**, arrow heads). Peripheral loop was defined as looping vessels in the periphery branching into vascular arcades between the vessel termini, and capillary fringe was defined as a fine peripheral network or flush of vessels on the margin of the CNV.

**Figure 6:**
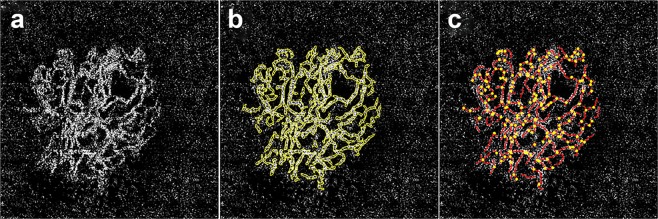
Optical coherence tomographic angiography (OCTA) image of exudative age-related macular degeneration in the “outer retina” (**a**). Outline of a neovascular lesion (**b**) and skeletonization (**c**) to calculate area, total length, and number of junctions of a neovascular lesion using semi-automated software.

The OCTA images were analyzed at least 24 months after the initiation of anti-VEGF treatment, when the clinical activity of the CNV lesion was most stable. If multiple OCTA images were available at different visits, the highest quality images with few artifacts were used for the analysis. The OCTA parameters included the morphologic patterns of CNV, presence of peripheral loop, and capillary fringe. The morphologic patterns of the CNV lesions were classified into two categories: CNV with a ‘closed circuit,’ defined as a presence of anastomotic vessel bounding the outer border of the vascular lesion for more than 50% of the entire CNV margin, and with an ‘open circuit,’ which includes anastomotic vessel for less than 50% (Fig. 5a,b). Peripheral loop was defined as looping vessels in the periphery branching into vascular arcades between the vessel termini, and capillary fringe was defined as a fine peripheral network or flush of vessels that were not individually resolved on OCTA (Fig. 5c,d). OCTA images were also analyzed for quantitative features such as area of CNV, total length of CNV, number of CNV junctions, and number of CNV end points using semi-automated, validated, open source software (Angiotool 0.5a, https://ccrod.cancer.gov/confluence/display/ROB2) (Fig. 6). All measurements were taken by two independent, masked retina specialists (K.B. and Y.K.S.). Disagreements over readings were resolved by open adjudication between observers.

### Measurements

We recorded patient age (years), sex, BCVA, lens status, and type of CNV at the time of the first angiographic examination. During follow-up visits at 3, 6, 12, and 24 months after initial treatment, all patients underwent BCVA measurement, dilated fundus examination, and SD-OCT. All treatments were recorded at each visit, along with the BCVA and measurements of CST of the macula. The following SD-OCT parameters were evaluated at baseline: the presence of SRF, IRC, and subfoveal choroidal thickness. In terms of OCTA parameters, the pattern of CNV, presence of peripheral loop, and capillary fringe, as well as quantitative parameters including the area of CNV, total length of CNV, number of CNV junctions, and number of CNV end points were also analyzed.

### Data analyses

The cohort of eyes with neovascular AMD was divided into two groups depending on individualized treatment response: the stop group, which met the cessation criteria of the TES protocol and did not show recurrence for more than 24 weeks without treatment until the last visit vs. the non-stop group, which required maintenance of injections due to recurrence after cessation of treatment or persistence of fluid. Measurements were compared between the two groups. Statistical analyses were executed using SAS version 9.4 (SAS Institute, Cary, NC, USA) and R 3.5.1 (http://www.R-project.org, Vienna, Austria). Inter-observer agreement regarding the morphologic features of CNV on OCTA was evaluated using the intraclass correlation coefficient (ICC) value. The best-corrected visual acuity was converted into LogMAR units prior to analysis. The chi-square test was used to compare categorical variables, while the Wilcoxon rank sum test and t-test were used for continuous variables. Appropriate parametric analyses were performed when the data were normally distributed. Stepwise multivariable correlation analysis was performed to identify correlations with discontinuation of treatment. P-values less than 0.05 were considered statistically significant.

### Statistical assistance

The authors thank the Medical Research Collaborating Center at Samsung Medical Center for help with statistical analyses.

## Supplementary information

Supplemental table 1
