# Supplementary material for: Predictors of neovascular activity during neovascular age-related macular degeneration treatment based on optical coherence tomography angiography
Source: Sci Rep. 2019 Dec 17;9:19240. doi: 10.1038/s41598-019-55871-8 (PMC6917758; doi:10.1038/s41598-019-55871-8)
Supplement: Supplementary file 1 — Supplemental table 1 [file 41598_2019_55871_MOESM1_ESM.docx]

**Predictors of neovascular activity during neovascular age-related macular degeneration treatment based on optical coherence tomography angiography**

Kunho Bae,^1,2^ Hyo Jung Kim,^2^ Yong Kyun Shin,^2^ Se Woong Kang^2^

**Institutional affiliations:**

^1^Department of Ophthalmology, Dongguk University, Ilsan Hospital, Goyang, South Korea

^2^Department of Ophthalmology, Samsung Medical Center, Sungkyunkwan University School of Medicine, Seoul, Korea

**Supplemental Table 1.** Optical coherence tomography angiography characteristics of patients with age-related macular degeneration according to the subtype of choroidal neovascularization.

| **Parameters** | **Typical AMD** | | | **PCV** | | |
| --- | --- | --- | --- | --- | --- | --- |
|  | Stop group (n =17) | Non-stop group (n =30) | p*-*value | Stop group (n =27) | Non-stop group (n =60) | p*-*value |
| Area of CNV, mm^2^ | 0.50 | 0.64 | .317 | 0.55 | 0.82 | .019 |
| Total length of CNV, mm | 13.3 | 17.3 | .281 | 14.7 | 21.6 | .018 |
| CNV junctions, No. | 72.5 | 89.9 | .431 | 73.1 | 110.6 | .016 |
| CNV end points, No. | 49.1 | 67.0 | .164 | 57.1 | 79.8 | .021 |
| Undetectable CNV | 12 (70.6%) | 0 | <.001 | 5 (18.5%) | 0 | <.001 |
| Pattern of CNV |  |  | <.001 |  |  | <.001 |
| Open-circuit | 5 (29.4%) | 9 (30.0%) |  | 16 (59.3%) | 24 (40.0%) |  |
| Closed-circuit | 0 | 21 (70.0%) |  | 6 (22.2%) | 36 (60.0%) |  |
| Peripheral loop | 1 (5.9%) | 17 (56.7%) | .001 | 7 (25.9%) | 46 (76.7%) | <.001 |
| Capillary fringe | 4 (23.5%) | 20 (66.7%) | .006 | 16 (59.3%) | 46 (76.7%) | .126 |

AMD, age-related macular degeneration; PCV, polypoidal choroidal vasculopathy; CNV, choroidal neovascularization.

Continuous variables are reported as mean values. All other data are n (%).
